# Religious values and confidence in science: Perceived tensions and common ground

**DOI:** 10.1371/journal.pone.0332477

**Published:** 2025-09-19

**Authors:** Isabelle Freiling, Michael A. Cacciatore, Meaghan McKasy

**Affiliations:** 1 Department of Communication, University of Utah, Salt Lake City, Utah, United States of America; 2 Grady College of Journalism and Mass Communication, University of Georgia, Athens, Georgia, United States of America; RMIT University, VIET NAM

## Abstract

While confidence in science is high compared to other institutions, many Americans question whether scientists share their values, including religious ones. Narratives surrounding science and religion often focus on a conflict between the two, but religious people, especially, tend to see their religious views as in line with science. Using survey data of two probability samples representative of U.S. adults, we examine how religious views, including perceptions of conflict and harmony between science and religion, predict confidence in science. We found that while general perceptions of religion and science as conflicting negatively predicted confidence in science, when individuals think religion endorses protecting the planet, their confidence in science was higher. The results suggest that attitudes toward religion and science are more nuanced than is often acknowledged and that audiences can be confident in science while holding strong religious beliefs. Further, the work suggests that finding and highlighting common ground between religion and science is a potentially promising avenue for cultivating confidence in science.

## Introduction

The public has a balanced view of science and technology that has been stable for over 60 years [1]. These broad attitudes toward science include both a dimension of confidence in science, or benefit perceptions of science, and a dimension representing concerns. A review of several survey datasets on attitudes toward science identified that “many of the surveyed U.S. public question the extent to which scientists share their values or overcome personal biases when presenting conclusions” [2]. A National Academies of Sciences, Engineering, and Medicine report suggests that those personal values can include religious views [3]. The following work focuses specifically on confidence in science. Confidence in science has been shown to be high when compared to other institutions [4], but it has decreased since the COVID-19 pandemic began in 2020 [2].

In discussions about science and religion, there is often a perception of tension [5–8]. For instance, Pew Research Center data [9,10] show that in 2024 half of Americans perceive science and religion as being mostly in conflict. However, that same survey also showed that those who are most likely to see a conflict are less-religious individuals. These realities suggest that, in something akin to the third-person effect, individuals are more likely to feel that the religion-science conflict exists for others but not for themselves, indicating that the perceived conflict could be overcome. It should be noted here that the U.S. seems to be an outlier when compared to other countries. Wellcome Global Monitor data show that across 140 countries only 29% of respondents say science has ever disagreed with the teachings of their religion, while in North America 57% say so [11].

As a secular nation that guaranteed religious freedom at its founding [12], the United States became a home for many religious practices fleeing persecution in other countries. Accordingly, several religious ideologies with unique worship practices proliferate in the United States today and, according to a 2023 Gallup survey, almost half (47%) of Americans identify as religious [13]. Interestingly, that percentage is lower than the percentage of people identifying as Christian in 2024, according to Pew Research Center data: 63 percent identify as Christian, with about six percent identifying as members of non-Christian religions [9]. Given the role religious views can play in confidence in science, coupled with the perceived tension between science and religion, as well as the fact that many Americans identify as religious or at least as members of a religion, this paper uses two probability samples of U.S. residents to examine how religiosity and perceptions of conflict between science and religion predict confidence in science. It should be noted here that we focus on religiosity in terms of importance of religion in one’s life, as this captures (non)religiosity for everyone, instead of on religious fundamentalism, which is applicable to a much smaller population. In Study 1, we examine more general religious-moral values as another predictor. Study 2 replicates the basic model (predicting confidence in science with religiosity and perceptions of conflict) and adds to that a perception of religion endorsing protecting the planet (which is in line with what the science on the issue suggests we should do), which is expected to increase confidence in science. By replicating across two independent, large probability samples, this study uniquely moves beyond isolated findings—a common limitation of solitary quota-based samples—to provide stronger empirical evidence for observable relationships.

## The relationship between science and religion

Perhaps the most influential work focusing on the relationship between religion and science is that of Barbour [6,7], who identified a four-category typology (*conflict, independence, dialogue,* and *integration*) of the ways science and religion might relate to each other. The first category of his typology argues that religion and science conflict, as exemplified by heated debates about whether and how evolution should be taught in schools or when noted atheists, like Richard Dawkins, speak aggressively about the incompatibility of scientific and religious beliefs.

The second category suggests science and religion operate independently of each other, due in large part to the differences in approaches to understanding (evidence-based versus faith-based, respectively) between the two [6,7,14]. Some have argued that the independence category can be an effective way of avoiding conflict between a scientific and religious way of viewing the world [15].

The final two categories in Barbour’s typology are more focused on the compatibility or similarities between science and religion. The dialogue perspective acknowledges that science can be helpful to religion and religion can be helpful to science—that while the two are distinct, they also overlap in important ways. It argues that people often turn to both science and religion when seeking answers to humanity’s big questions [6,7,14] and that each can be valuable in arriving at an understanding of truth. These might include making sense of our existence as a species or explaining the origins of the universe. The dialogue perspective appears consistent with studies that have revealed that people are often motivated to turn to careers in science to satisfy one of the central tenets of virtually all the world’s religions—namely, to help make the world a better place [16].

Finally, the integration perspective sees religion and science as operating in tandem or as complements to each other [6,7,14]. While similar to the dialogue perspective, integration pushes the conversation between religion and science slightly further, arguing that the best way to understand the world is by integrating the two to arrive at a more complete picture or understanding. Based on these categories, the literature review first elaborates on the tension between science and religion before moving on to a discussion of the ways science and religion share common ground.

### The tension between science and religion

There is a common perception of tension between science and religion [8], yet this is not a universal understanding. Some individuals who hold religious beliefs see science and religion as compatible or even unrelated parallel concepts [17], even among Christians [9]. One way in which these notions vary is based on how central religion is to your life. While half of Americans identify a broad tension between religion and science [9], when questioned about their individual religious beliefs and science, they are much less likely to identify a clash [10].

The perceived conflict between science and religion often focuses on abstract and heuristic considerations of science and religion, frequently exacerbated by the media, rather than empirical issues between the two [5]. In fact, religious people’s distrust of science often comes from the perception that scientific agendas are biased in a way that threatens religious values [18,19]. For many religious individuals, even if they do not broadly distrust science, they are aware of anti-religious science or scientist stereotypes and may help perpetuate those stereotypes through actions and conversations [20]. There exists substantial evidence that individuals are motivated to use information processing strategies that align with and lead to their desired conclusion [21]. Therefore, if there is a perceived tension between religion and science, an individual may preserve or even strengthen that conflict through their motivated reasoning to support their religious ideology.

Additionally, when someone feels that information or behaviors do not align with their personal values or beliefs, they often experience cognitive dissonance and will work to remove the discomfort of contradiction [22]. That could take many forms in the dynamic between science and religion. The formation of an ingroup versus outgroup social identity mentality may explain some of this tension, as perceived threats to ingroup identity can moderate an individual’s trust in science [23]. In the context of video gaming, for instance, gamers were more likely to publicly discredit scientific research that threatened their group identity than research that did not [24]. Further, nonreligious individuals have been shown to stereotype religious individuals as unscientific and less intelligent [25], which can lead to more threat perceptions by the outgroup for religious individuals.

Clearly, several factors influence the ways that individuals process information to form attitudes and influence behaviors. As religion is a part of religious people’s identities, religious truisms and value predispositions can act as heuristic cues to aid in complex decision-making [26]. In contrast, scientific thinking is often more systematic as it requires a baseline level of skill and cognition that is not always as accessible to people and can require more effort. In line with dual processing models, such as the heuristic systematic model [27], people tend to rely more often on the heuristic way of processing information. As such, when science and religion seemingly conflict, science may face greater difficulty in emerging as the prevailing insight if individuals rely on a religious heuristic cue that might trigger emotions [19].

Finally, religious teachings often provide a moral framework that guides a follower’s behavior with adherence to ethical principles and guidelines. This framework formulates the understanding of what is morally right or wrong. In fact, many Americans consider a belief in God to be a necessary condition for morality [25]. Yet, exposure to science itself can influence moral norms and behaviors [28]. Indeed, several scientific advancements central to research agendas, such as embryonic stem cell research or reproductive genetics, appear to conflict with the moral understanding of many religious traditions [18].

Beyond the importance of religion in life and the conflict people may see between science and religion, the religious values that people hold represent another part of their religiosity that may be associated with their confidence in science. While moral values can encompass many more values that guide our everyday thinking and behavior [29], some that specifically match religious teachings are about the immorality of abortion, divorce, sex before marriage, and homosexuality [29–31]. Given that atheism is more common among scientists than the general public [19], scientists may be perceived as being less likely to hold those same religious and moral values. Nonetheless, those values may be very important to religious people and close to their religious identity, and might thus be considered regarding their trust in science.

Religion and science exist in culturally and socially relevant domains and, as such, come into contact. As previously discussed, there are several dimensions of religion where this contact may influence a perceived conflict between religion and science: epistemological knowledge formation, social practices, and moral frameworks. We assume that when the religious identity plays a bigger role in someone’s life and they are getting more guidance from religion in their life, they would have less confidence in science:

*H1: The more guidance in life people get from religion, the less confidence they have in science.*

Additionally, a religious framework can guide an individual’s moral understanding of right and wrong, often over issues that may stir strong value-based beliefs (i.e., abortion, homosexuality, etc.). We therefore assume:

*H2: The more people agree with religious-moral values, the less confidence they have in science.*

Given that we know less about the relationship between perceiving a conflict between science and religion and confidence in science, we ask:

*RQ1: How is viewing science and religion as conflicting associated with confidence in science?*

### Highlighting common ground between science and religion

The dialogue and integration categories of Barbour’s [6,7] typology highlight that it is possible to find common ground about the importance or role of science in society between those of varying religious beliefs. This might involve, for example, providing the right cues, context, or framing when discussing the role of science in addressing societal issues, such as appealing to a resonant religious chord among those who more strongly identify as people of faith. One method of doing so is the framing of environmental topics as appealing to religious values of environmental protection, or as it is sometimes also called, environmental stewardship. There is no single definition of environmental stewardship across religious ideologies; however, it generally refers to humans being entrusted with the environment, and having more obligation to care for it as it is God’s creation [32]. Of course, religious individuals are not a monolith, and disagreements abound within a given religion and even within denominations about the degree to which humans should be responsible for the environment. Nevertheless, the idea of environmental stewardship has been outlined as a promising avenue for preaching pro-environmental messages to religious audiences.

The early evidence for whether priming, framing, or otherwise drawing attention to one’s religious values is an appropriate means for persuading audiences to take pro-environmental actions or adopt pro-environmental attitudes is best described as mixed. Some work has identified stronger feelings of environmental mastery among religious individuals than nonreligious individuals [33], with further studies suggesting that stronger belief in the Bible, as well as higher scores on religious commitment and adherence to religious tradition, are all negatively associated with environmental concern [34,35]. Conversely, work by Shaiko [36] found that while those with a religious affiliation were indeed more likely to hold views of human mastery over the environment, such feelings need not necessarily translate into lower levels of environmental concern, and instead, could be associated with more of a stewardship orientation toward nature.

Such contradictory findings concerning religion’s relationship with the environment is speculated to be the result of inconsistencies in the specific religious values that may have been top-of-mind or salient at the time the study participants were expressing their opinions [37], an explanation that would be consistent with the attitude accessibility literature [38,39]. To test this explanation, Goldberg and colleagues [40] utilized a two-study approach specifically focused on the social identity of Christians. The first study identified a desire “to protect God’s creation” as a top motivator Christians mentioned for wanting to mitigate global warming. In the second study, participants were randomly assigned to receive different messages, including a pro-environmental stewardship message that also highlighted the participant’s Christian identity. The experiment revealed that a “stewardship” frame could increase climate change belief and pro-environmental attitudes among Christian Americans by increasing their perception that environmental protection was a religious issue, including one that other in-group Christian members also believed to be important [40]. In sum, the authors argued that a communication approach built around one’s religious identity can be an effective strategy for encouraging pro-environmental behaviors among some religious audiences.

Given that more recent research indicates that framing messages about protecting the environment is something that can resonate with religious audiences, we propose the following hypothesis:

*H3: The perception that religion endorses protecting the planet is positively associated with confidence in science.*

### Demographic controls

Basic demographics have also been found to correlate with perceptions of science, although the patterns often vary across different scientific topics. For example, Democrats and politically liberal audiences generally score higher on measures of support for science than Republicans and conservatives [41,42]; however, this pattern is reversed when a topic like nuclear power is the science and technology under investigation [43]. Similarly, while women have generally been found to show greater concern or perceptions of risk toward scientific research and new technologies [44], a focus on measures like trust or confidence can paint a decidedly different picture about who is more supportive of science [44]. Indeed, patterns related to gender or political ideology often become null, or even flip when factors like the race/ethnicity of the men or women being queried are included in the analysis [42,45]. While basic demographics are not at the heart of our current work, we nonetheless understand the need to control for such factors, and do so by including measures of sex, age, education, race/ethnicity, and political ideology in our analyses.

### Study 1

Study 1 focuses on the tension between science and religion and examines how the importance of religion, whether science and religion are perceived to be in conflict, and religious-moral values predict confidence in science (H1, RQ1, H2).

#### Methods

We use data from the World Value Survey (WVS) wave 7 of adults aged 18 and older from the U.S. for Study 1, which was collected between April 28 and May 31, 2017. Data were accessed on September 9, 2023. We focused the analysis on this subgroup (U.S. adults) to allow for comparison between studies since participants in Study 2 were from the U.S. Further, we recognize that religions differ widely around the world, and this focus allows us to comment on the particular mechanism at play between science and religion in the U.S. WVS survey administrators followed ethical standards, and respondents provided either written or verbal (via telephone) informed consent before participating in this study, depending on the format they would take the survey in (computer-assisted web interviews vs. computer-assisted telephone interviews).

#### Participants.

Respondents’ (*N* = 2,596) age ranged from 18 to 90 years, and was on average 43.42 years (*SD* = 16.31) with 53.5 percent identifying as male and 46.5 percent as female. The median education was short-cycle tertiary education, which is an associate degree (education was measured with ISCED [International Standard Classification for Education] 2011 codes, as used by the United Nations [UN] and the United Nations Educational, Scientific and Cultural Organization [UNESCO]. Those codes range from early childhood education/ no education [coded 0] to doctoral or equivalent [coded 8], and represent the respondents’ highest level of education). In terms of race/ethnicity, 66.1 percent of respondents self-identified as White, non-Hispanic, 8.1 percent as Black, non-Hispanic, 17.4 percent as Hispanic, 4.7 percent as other, non-Hispanic, and 3.3 percent as two plus, non-Hispanic.

#### Measures.

*Confidence in science’s* ability to positively impact the world was measured with how much respondents agree or disagree (1 = completely disagree, 10 = completely agree) with the following statements [adapted from 46]: (a) Science and technology are making our lives healthier, easier, and more comfortable (*M* = 7.25, *SD* = 2.32). (b) Because of science and technology, there will be more opportunities for the next generation (*M* = 7.15, *SD* = 2.32). We merged the two variables (*r* = .66) into a mean index (1–10; *M* = 7.20, *SD* = 2.12).

*Importance of religion* was measured with “For each of the following, indicate how important it is in your life: Religion” (1 = not at all important, 4 = very important; *M* = 2.70, *SD* = 1.13).

*Conflict of religion and science* was measured with respondents’ agreement (1 = strongly disagree, 4 = strongly agree) with the statement “Whenever science and religion conflict, religion is always right” (*M* = 2.13, *SD* = 0.99).

*Religious-moral values* were measured along with a list of several other values with “Please tell me for each of the following actions whether you think it can always be justified, never be justified, or something in between, using this card” (1 = never justifiable, 10 = always justifiable). From the list of values provided to respondents in the WVS, we focus on those that are clearly related to religious moral values: (a) homosexuality (*M* = 6.50, *SD* = 3.41), (b) abortion (*M* = 5.07, *SD* = 3.00), (c) divorce (*M* = 6.60, *SD* = 2.45), and (d) sex before marriage (*M* = 6.72, *SD* = 2.93). Reliability between the four items is good (Cronbach’s *α* = .85), and a confirmatory factor analysis using Varimax rotation shows all items loading on one factor. Before merging the items into an index, we recoded them so that high values for the index represent more agreement with religious-moral values (1–10, *M* = 4.79, *SD* = 2.46).

*Controls* We controlled for sex, age, education, race, and political ideology. For descriptives of sex, age, education, and race, see the section on Participants above. *Sex* was measured in binary (1 = male, 2 = female). *Race/ethnicity* was measured with White, non-Hispanic; Black, non-Hispanic; other, non-Hispanic; Hispanic; and two plus, non-Hispanic. We included dummy codes for Black, Hispanic, and other (non-Hispanic other races and two plus races) in our analysis, leaving White as the reference category. *Political ideology* was measured with “In political matters, people talk of ‘the left’ and ‘the right.’ How would you place your views on this scale, generally speaking?” on a scale from 1 = left to 10 = right (*M* = 5.22, *SD* = 2.52).

#### Analyses.

The analyses were conducted in SPSS version 29. To match the sample to Census data, we applied weights to the sample before conducting a hierarchical regression analysis predicting confidence in science. In the first step, we added the control variables. In the second step, we added the variables importance of religion and conflict of religion and science, which allows for better comparison of the results of both studies. We report results from this model for H1 and RQ1. In the last step, we entered religious-moral values, a construct that Study 2 does not cover.

## Results

For an overview of the results, see Table 1. We report unstandardized regression coefficients (*B*), their 95% confidence intervals (*CI*), as well as—for better comparison across both studies—effect sizes (semi-partial correlations, *r*_*sp*_) [47]. VIF (range: 1.05–2.08) and tolerance values (range: .48 −.95) indicate no issues with multicollinearity (for a table with bivariate correlations between the key independent variables and the dependent variable, see S2 Table, and for a table with VIF and tolerance values, see S3 Table in Supporting Information). Model 1 had an adjusted R square of .07 (*p* < . 001), model 2 of .10 (*p* < . 001), and model 3 of .11 (*p* < . 001). A post hoc power analysis shows a power of 1.0.

**Table 1:** Hierarchical regressions predicting confidence in science’s ability to positively impact the world in Study 1.

	Model 1			Model 2			Model 3		
	*B*	*CI*	*r*_sp_	*B*	*CI*	*r*_sp_	*B*	*CI*	*r*_sp_
(Constant)	6.49	[5.96; 7.01]		7.10	[6.56; 7.64]		7.31	[6.77; 7.84]	
Sex	−0.16	[-0.33; 0.01]	−.04	−0.06	[-0.23; 0.11]	−.01	−0.14	[-0.31; 0.03]	−.03
Age	0.01	[0.01; 0.02]	.08	0.01	[0.01; 0.02]	.08	0.01	[0.01; 0.02]	.09
Education	0.21	[0.15; 0.26]	.15	0.16	[0.11; 0.22]	.11	0.14	[0.09; 0.20]	.10
Black (reference category: White)	−0.70	[-0.96; -0.43]	−.10	−0.43	[-0.70; -0.16]	−.06	−0.41	[-0.68; -0.14]	−.06
Hispanic (reference category: White)	0.09	[-0.15; 0.33]	.01	0.17	[-0.06; 0.41]	.03	0.18	[-0.06; 0.41]	.03
Other races (reference category: White)	0.25	[-0.06; 0.56]	.03	0.32	[0.01; 0.62]	.04	0.39	[0.09; 0.69]	.05
Ideology (conservative)	−0.10	[-0.14; -.0.07]	−.12	−0.05	[-0.08; -0.01]	−.05	−0.02	[-0.05; 0.02]	.02
Importance of religion				0.02	[-0.08; 0.12]	.01	0.12	[0.02; 0.22]	.04
Conflict of religion and science: Whenever science and religion conflict, religion is always right.				−0.43	[-0.55; -0.32]	−.14	−0.32	[-0.44; -0.20]	−.10
Religious-moral values							−0.15	[-0.20; -.0.11]	−.13
Adjusted R square	.07 (*p* < .001)			.10 (*p* < .001)			.11 (*p* < .001)		
*N = *2,455									

Table shows unstandardized regression coefficients (*B*), their 95% confidence intervals (*CI*), and effect sizes, semi-partial correlations (*r*_sp_).

Importance of religion in the life of people did not predict their confidence in science in model 2 (*B* = 0.02, CI [−0.08; 0.12]), not supporting H1. Regarding RQ1, the more people viewed religion and science as conflicting (with religion being always right in those cases), the less confidence they had in science (*B* = −0.43, CI [−0.55; −0.32], *r*_*sp*_ = −.14). The more people agreed with religious-moral values (model 3), the less confidence they had in science (*B* = −0.15, CI [−0.20; -.0.11], *r*_*sp*_ = −.13), supporting H2.

## Discussion

The results suggest that the relationships between one’s religious beliefs and attitudes toward science are more nuanced than is often depicted in discussions of the topic. Most notably, merely being religious is not synonymous with a lack of confidence in science. Our results showed no significant relationship between the importance one ascribes to their religion and confidence in science. However, when one’s feelings toward religion are manifested in a belief that religion and science are incompatible or when one’s religious beliefs include absolutist views about the immorality of practices like homosexuality, abortion, divorce, and sex before marriage, confidence in science declines. Before delving further into these findings, we turn to our second study, which looks at how highlighting different attributes of science might make science more compatible with religious views.

## Study 2

Study 2 is a conceptual replication of H1 and RQ1 of Study 1 that also examines how perceiving religion as endorsing to protect the planet (H3) predicts confidence in science. The data used for the secondary analysis in Study 2 were collected as part of a larger project on public opinion regarding biomanufacturing.

### Methods

The survey was fielded by the National Opinion Research Center (NORC) at the University of Chicago with their probability-based AmeriSpeak panel of U.S. adults aged 18 and older between November 2 and November 10, 2023. Participants received compensation for their time which equals $3. Study 2 was approved by the Institutional Review Board of NORC at the University of Chicago. The survey was completed online with consent obtained digitally via a “consent” button prior to entering the survey.

#### Participants.

Overall, 3,998 panelists were contacted, with 806 completing the survey, leading to a survey completion rate of 20.2 percent. The gender of the respondents was evenly split (49.6% of the respondents were male, with 50.4% female). Respondents’ age ranged from 18 to 91 years with an average age of 49.69 years (*SD* = 17.69). Education was measured on a 5-point scale (1 = less than high school, 2 = high school or equivalent, 3 = some college/ associates degree, 4 = Bachelor’s degree, 5 = postgraduate study/ professional degree; Median: some college/ associates degree). In terms of race/ethnicity, 64.1 percent of respondents self-identified as White, non-Hispanic, 11.7 percent as Black, non-Hispanic, 17.4 percent as Hispanic, 1.7 percent as other, non-Hispanic, 2.2 percent as two plus, non-Hispanic, and 3.3 percent as Asian-Pacific Islander, non-Hispanic.

#### Measures.

*Confidence in science’s* ability to positively impact the world was measured by asking respondents how much they agree or disagree (1 = strongly disagree, 7 = strongly agree) with the following statements: (a) Science makes our lives easier and more comfortable (*M* = 5.13, *SD* = 1.36). (b) Because of science, there will be more opportunities for the next generation (*M* = 5.25, *SD* = 1.42). While this measurement is similar to the WVS measurement, we only focused on science and not on technology in this survey, and simplified the items. We merged the two variables (*r* = .73) into a mean index (1–7; *M* = 5.19, *SD* = 1.29).

*Importance of religion* was measured by asking respondents “To what extent do you look to the following for guidance on right and wrong in your everyday life?: Religion” (1 = no guidance at all, 7 = a great deal of guidance, *M* = 4.07, *SD* = 2.19).

*Conflict of religion and science* was measured with respondents’ agreement (1 = strongly disagree, 7 = strongly agree) to the following statement: “Science is incompatible with my religious views” (*M* = 2.65, *SD* = 1.73).

*Religion endorses protecting the planet* was measured by asking respondents about their agreement (1 = strongly disagree, 7 = strongly agree) with the following two statements: (a) My religious views emphasize our responsibility to protect the planet” (*M* = 4.27, *SD* = 1.80) and (b) “Science is one of the tools God gave us to protect the planet” (*M* = 4.61, *SD* = 1.72). We merged both items (*r* = .52) into a mean index (1–7; *M* = 4.44, *SD* = 1.53).

*Controls* We controlled for sex, age, education, race, and political ideology. For descriptives of sex, age, education, and race, see the section on Participants above. *Sex* was measured in binary (1 = male, 2 = female). We included dummy codes for Black, Hispanic, and other (non-Hispanic other races, non-Hispanic two plus races, and non-Hispanic Asian-Pacific Islander) in our analysis, leaving White as the reference category. *Political ideology* was measured with “The terms ‘liberal’ and ‘conservative’ may mean different things to people, depending on the kind of issue one is considering. In terms of (a) economic issues and (b) social issues, would you say you are…” on a scale from 1 = very liberal to 7 = very conservative (economic issues: *M* = 4.03, *SD* = 1.59, social issues: *M* = 3.78, *SD* = 1.67, *r* = .78). We merged both items into a mean index (1–7; *M* = 3.91, *SD* = 1.54).

#### Analyses.

Similar to Study 1, we applied weights to the sample before conducting a hierarchical regression analysis predicting confidence in science to match our sample to Census data. In the first step, we added the control variables, before entering in the second step the variables importance of religion and conflict of religion and science for a better comparison of the results across both studies. As in Study 1, we will thus report model 2 for results pertaining to H1 and RQ1. In the last step, we added the construct that Study 1 did not cover: religion endorsing the protection of the planet. The data of Study 2 can be found in Supporting Information file study_2_data.csv.

### Results

For an overview of the results of this study, see Table 2. Like for Study 1, we report unstandardized regression coefficients (*B*), their 95% confidence intervals (*CI*), as well as—for better comparison across both studies—effect sizes (semi-partial correlations, *r*_*sp*_) [47]. VIF (range: 1.02–1.59) and tolerance values (range: .63 −.98) indicate no issues with multicollinearity (for a table with bivariate correlations between the key independent variables and the dependent variable, see S4 Table, and for one with VIF and tolerance values, see S5 Table in Supporting Information). Model 1 had an adjusted R square of .11 (*p* < . 001), model 2 of .13 (*p* < . 001), and model 3 of .21 (*p* < . 001). A post hoc power analysis shows a power of 1.0.

**Table 2:** Hierarchical regressions predicting confidence in science’s ability to positively impact the world in Study 2.

	Model 1			Model 2			Model 3		
	*B*	*CI*	*r*_sp_	*B*	*CI*	*r*_sp_	*B*	*CI*	*r*_sp_
(Constant)	5.90	[5.44; 6.36]		6.00	[5.54; 6.46]		5.05	[4.56; 5.53]	
Sex	−0.41	[-0.58; -0.23]	−.16	−0.36	[-0.53; -0.19]	−.14	−0.34	[-0.50; -0.18]	−.13
Age	0.01	[0.00; 0.01]	.08	0.01	[0.00; 0.01]	.08	0.01	[0.00; 0.01]	.10
Education	0.13	[0.05; 0.20]	.12	0.11	[0.04; 0.19]	.10	0.10	[0.03; 0.17]	.09
Black (reference category: White)	0.27	[0.00; 0.54]	.07	0.43	[0.15; 0.71]	.10	0.40	[0.14; 0.67]	.09
Hispanic (reference category: White)	−0.15	[-0.38; 0.09]	−.04	−0.05	[-0.29; 0.19]	−.01	−0.02	[-0.25; 0.20]	−.01
Other races (reference category: White)	−0.16	[-0.47; 0.15]	−.03	−0.12	[-0.43; 0.19]	−.03	−0.04	[-0.34; 0.25]	−.01
Ideology (conservative)	−0.21	[-0.26; -.0.15]	−.24	−0.18	[-0.24; -.0.11]	−.18	−0.16	[-0.22; -0.10]	−.17
Importance of religion				0.01	[-0.04; 0.05]	.01	−0.08	[-0.12; -0.03]	−.10
Conflict of religion and science: Science is incompatible with my religious views.				−0.12	[-0.17; -.0.07]	−.15	−0.11	[-0.16; -0.06]	−.14
Religion endorses protecting the planet							0.26	[0.20; 0.31]	.28
Adjusted R square	.11 (*p* < .001)			.13 (*p* < .001)			.21 (*p* < .001)		
*N = *785									

Table shows unstandardized regression coefficients (*B*), their 95% confidence intervals (*CI*), and effect sizes, semi-partial correlations (*r*_sp_).

As in Study 1, importance of religion in the life of people did not predict their confidence in science in Study 2’s model 2 (*B* = 0.01, CI [−0.04; 0.05]), not supporting H1. Similar to Study 1, the more people viewed their religion and science as incompatible, the less confidence they had in science (**B* *= −0.12, CI [−0.17; -.0.07], *r*_*sp*_ = −.15), answering RQ1.

When people perceive religion as endorsing the protection of the planet (model 3), people report a higher confidence in science (**B* *= 0.26, CI [0.20; 0.31], *r*_*sp*_ = .28), supporting H3. Interestingly, when including this measure in the model, which represents to some extent the compatibility between religion and science regarding protecting the planet, the importance of religion in people’s lives became a significant predictor of confidence in science. In other words, the more important religion was in people’s lives, the less confidence they had in science (**B* *= −0.08, CI [−0.12; -.0.03], *r*_*sp*_ = −.10).

### Discussion

Overall, we were able to replicate the findings of Study 1 regarding RQ1 in this study, and when running the model that includes the same conceptual variables as Study 1 (model 2), we were also able to replicate the findings regarding H1. However, given the different findings when including the final variable in the model, it seems that the null findings regarding H1 might not be completely robust, and instead, depend on what other variables are being controlled for in the analysis. We discuss potential reasons for that as well as elaborate on our overall findings and limitations to the present work in the General discussion that follows.

## General discussion

Before discussing both studies together, there are several limitations to consider. First, our analysis does not distinguish between different religious affiliations or denominations within religious affiliations. It may be that nonreligious individuals view these topics quite differently and are disproportionately driving the findings outlined here. For example, while overall in the U.S. 50% of individuals think science and religion are often in conflict, two-thirds (68%) of religiously unaffiliated people perceive science and religion to be often in conflict [9,10].

Second, not all religious individuals perceive a conflict between religion and science. In fact, only about 42% of religious people do [9]. While we based H1 and H2 not only on the conflict perception, but also on social identity research and scientists being generally perceived as being less religious, we ran a post hoc interaction between importance of religion and perceived conflict between science and religion in both studies to examine whether the relationship between importance of religion and confidence in science (H1) is moderated by individuals actually perceiving a conflict. In Study 1, we found such an interaction effect (**B* *= 0.19, CI [0.09; 0.29], *r*_*sp*_ = .07), but the interaction plot (S1 Fig in Supporting Information) does not offer a picture in line with that. In fact, it seems like individuals for whom religion is not at all important and who perceive religion to be always right in a conflict to have the lowest confidence in science. Examining cross tabulations of the importance of religion and conflict between religion and science showed that these individuals were a few outliers. Additionally, Study 2 did not replicate the significant interaction of Study 1, which could be due to the different wording in the item measuring conflict between science and religion. Thus, the significant interaction in Study 1 is not only theoretically odd to interpret and likely caused by a few outliers, but also not a robust finding. We also ran a post hoc interaction between religious-moral values and perceived conflict between science and religion in Study 1 to examine whether the relationship between religious-moral values and confidence in science (H1) is moderated by individuals actually perceiving a conflict. This interaction was not significant. Future research might be able to shed more light on whether the interaction with importance of religion and perceived conflict found in Study 1 was, in fact, not robust or whether the result does depend on highlighting in the conflict item not only that there is a conflict between science and religion, but also that when there is a conflict it would be religion which is always right.

Additionally, while we used WVS survey data in Study 1, we only focused on the subsample the WVS had on Americans in our analysis to keep the population the same across both studies for the replication. To examine the above point about the potential interaction cross-nationally, future research could also use the WVS dataset and examine all included countries. By doing that and perhaps sorting countries per their average perceived conflict, such a study could test whether the positive relationship we found for H1 might only hold in countries where perceived conflict is high.

Third, H3 posits a positive association between the perception that religion endorses environmental protection and confidence in science. Recent Pew Research Center findings support this approach, indicating that nearly half of U.S. adults believe that science and religion are compatible, with this sense of compatibility increasing among more religious individuals [9]. We recognize, however, that this positive association may not hold true for all individuals. Some may, in fact, associate science with environmental degradation, citing issues such as pollution and resource depletion. This association may stem from historical moments of scientific advancements, like the Industrial Revolution, which resulted in significant environmental harm. As H3 primarily focuses on the areas of compatibility and common ground between science and religion, it may not fully capture the perspectives of those who perceive a negative or conflicting association. Future research could include a measure of the specific attitudes toward science and its relation to the environment.

It should also be acknowledged that our analyses are based on two snapshots in time. While relying on two data collections is an improvement over the vast majority of studies which tend to rely on a single data collection, the two data collections employed here varied in terms of sample size and time frame, with Study 1 based on data collected prior to the COVID-19 pandemic, in 2017, and Study 2 being based on data from the end of 2023. Regardless, it is worth noting that the general consistency in patterns between the two data collections suggests the identified relationships are manifesting among American audiences. At the same time, some of the discrepancies in findings—such as the differences in significance between sex and confidence in science and the reversed coefficient among the Black participants in our sample—may be the result of the unique data collections and time when each were collected.

While every effort was made to ensure the equivalence of the two data analyses, we are relying on secondary data for Study 1 and 2, meaning that the two datasets are not wholly compatible and often included slightly different wordings for similar concepts. Most notably, the WVS dataset (Study 1) probed the conflict between religion and science by asking about agreement with the statement, “Whenever science and religion conflict, religion is always right,” while the dataset used in Study 2 utilized agreement with the following statement: “Science is incompatible with my religious views.” It is possible that the slightly different wordings prompted unique thoughts and considerations in respondents, which similar items seem to have prompted in Pew Research Center data [10]. While the data used in this manuscript are also secondary for Study 2, we were responsible for building that survey instrument, which was not the case for Study 1. Thus, in Study 1, we had no control over the item being about religion being always right whenever there is a conflict between science and religion. In fact, it could be that people perceive a conflict, but would not agree that religion would be right in that conflict (and instead would think science is right). Thus, when we crafted the statements for the survey that Study 2 uses, we decided to change that item to a mere incompatibility, without including what would be right in a situation where science and religion conflict. This change in the statement then also allowed us in this manuscript to test whether it would lead to a difference in the relationship between conflict perceptions and confidence in science. However, the consistency of the two items with our key dependent variable suggests this was not the case here as we were able to conceptually replicate the relationship.

Relatedly, we relied on single-item measures for importance of religion and conflict of religion and science in both studies. While single-item measures are commonly used for this type of research [see, for example, for religiosity in science communication research: 48, 49, 50] single-item measures are, of course, not ideal. Future research using multiple items for these constructs could add more nuance.

Finally, the response rate in Study 2 was 20.2%, which may raise concerns regarding non-response bias. However, the unweighted sample did not deviate substantively from the Census benchmark. Still, we used weighted data for our regression analysis to ensure an even better match to U.S. Census data (see S1 Table in Supporting Information).

Keeping those considerations in mind, our findings are notable for several reasons. First, interpreted positively, the findings suggest that the door is very much open for growing confidence in science among even the highly religious. The findings provide evidence that the general population may be responsive to the idea that science and religion can operate in tandem, as Barbour [6,7] suggests with his typology concerning the relationship between science and religion. Our results suggest that the oft-discussed tension between science and religion is not a zero-sum game, whereby placing a great deal of importance on religion necessarily means that confidence in science has to suffer. It appears people can simultaneously hold a strong need for religion alongside high confidence in science. Indeed, a similar pattern revealed itself when exploring the relationship between age and confidence in science across the two data collections. As one reviewer astutely noted, it is generally expected that one’s religiosity will increase as one ages, leading one to expect that older individuals might express lower confidence in science, rather than the higher confidence in science that our datasets revealed. We interpret this finding in a very practical manner, having to do with one’s mortality as one ages. As one gets older, (medical) science plays an arguably bigger role in day-to-day life as it becomes the chief means of staying healthy and prolonging life. We suspect it is only natural to want to trust or put faith in the scientific interventions that are seen as central to keeping one healthy. Perhaps the most obvious example of this comes from studies of vaccine-related attitudes and behaviors, which consistently show age to be among the most important predictors of compliance year after year [51].

Second, while topics like homosexuality, abortion, divorce, and sex before marriage do not appear to be obvious science topics, we expected the negative correlation we found between acceptance of these practices and public confidence in science because views on these issues are close to core religious teachings, while science and scientists are often perceived as nonreligious, thus potentially not sharing the same values on these topics. Perhaps these measures thus tap into a form of traditional religious thinking that is more in line with a fear of societal change, rather than something overtly tied to science. The good news for those hoping to strengthen public confidence in science is that such thinking is on the decline not only in the U.S. but in many parts of the world [52]. As one example, there has been an overall trend toward the liberalization of abortion laws, despite the reversal of Roe v. Wade by the U.S. Supreme Court [53]. For instance, since 2000, nearly 40 countries had altered their pre-existing abortion laws, with all but the U.S. and Nicaragua doing so by expanding women’s access to abortion [53].

Looking more attitudinally, in the U.S., the percentage of people who say abortion should be available for any reason reached 55% in 2022 (the last year with available data), the highest the GSS has recorded seen since it started posing the question, and almost double the low-point of 32% recorded in 1983 [54]. Similar patterns can be seen with the other issues explored in this analysis. For example, the GSS found that 45% of Americans agree that same-sex couples should have the right to marry, up from just 11% in 2004 [55], while also finding that the percentage who view sex before marriage as “always wrong” has been halved from 29% in 2002 to 14% in 2022 [56]. Lastly, when asked whether divorce should be made easier or more difficult to obtain, American public opinion has shifted from 24% claiming it should be made easier in 2006 to 42% feeling that way in 2018 (the last year the question was posed) [57]. Altogether, this suggests that either the influence of the church concerning broader societal questions may be softening, or that their approach to those topics is evolving. Regardless, it is reasonable to expect that younger audiences are more progressive on these topics (homosexuality, abortion, divorce, and sex before marriage), thereby reducing a significant barrier to public confidence in science. It will be interesting to see how such societal shifts are tied to thinking about emerging science that has been said to straddle ethical and moral boundaries, such as biomanufacturing and gene editing technologies, that some say allow scientists to play the role of God [58].

Third, the findings show that perceiving religion as endorsing a topic that is often associated with science—the protection and preservation of the natural environment—is positively associated with confidence in science. This idea is rather intuitive as it suggests recognizing common ground or harmony between science and religion can enhance the appeal of science to a more religious audience. Less certain is whether similar appeals focused on religious teachings, but in realms outside of the natural environment, might be used to further broaden the appeal of science to religious audiences. For instance, might appeals focused on helping the disadvantaged be used to push for different types of food science, like biotechnology or plant-based meat alternatives?

Fourth, while Study 2 largely replicated the key findings from Study 1, when including the perception that religion endorses the protection of the planet in the model, this altered the previously non-significant relationship between the importance of religion in people’s lives and scientific confidence. That is, the relationship turned from non-significant to negative, indicating that the more importance a respondent ascribed to religion, the lower their reported confidence in science. As we are not only controlling for the conflict category of Barbour’s [6,7] typology in model 3 (by including feelings of their religion and science being incompatible as we do in model 2), but also for the perception that religion endorses the protection of the planet, model 3 predicts with the variable of importance of religion only those parts of the construct of important of religion that do not overlap with conflict between science and religion and religion endorsing the protection of the planet.

All told, the conceptual replication provides robust evidence for patterns observed in the complex relationship between science and religion, which is generalizable to the broader American population—a crucial point given that a significant percentage identifies as religious. The present work demonstrates that attitudes toward religion and science are more nuanced than is often acknowledged and that audiences can be confident in science while holding strong religious beliefs. Further, the work suggests that finding common ground between religion and science—in this instance, viewing religion as endorsing the protection of the planet, which is in line with what science suggests—is a potentially promising avenue for cultivating confidence in science. Overall, the work highlights the promise of framing and messaging as important communication tools for building public confidence in science.

## Supporting information

S1 File Dataset for Study 2. The first row contains variable names, the second variable labels, and the third variable values, where applicable.
(CSV)

S1 Table Benchmark Comparisons of Study 2 Data. (DOCX)

S2 Table Bivariate Correlations for Key Independent Variables and Dependent Variable for Study 1. *** p < .001. (DOCX)

S3 Table VIF and Tolerance Values for Study 1. (DOCX)

S4 Table Bivariate Correlations for Key Independent Variables and Dependent Variable for Study 2. * p < .05, *** p < .001. (DOCX)

S5 Table VIF and Tolerance Values for Study 2. (DOCX)

S1 Fig Interaction Plot of Importance of Religion and Conflict of Religion and Science, Predicting Confidence in Science. (TIFF)
