# Supplementary material for: Religious values and confidence in science: Perceived tensions and common ground
Source: PLoS One. 2025 Sep 19;20(9):e0332477. doi: 10.1371/journal.pone.0332477 (PMC12448960; doi:10.1371/journal.pone.0332477)
Supplement: S1 Table — (DOCX) [file pone.0332477.s002.docx]

**S1 Table. Benchmark Comparisons of Study 2 Data.**

| **Demographic Category** | **Subcategory** | **Unweighted (%)** | **Weighted (%)** | **Benchmark (U.S. Census, %)** |
| --- | --- | --- | --- | --- |
| **Age** | 18 - 24 | 7.2 | 11.8 | 11.8 |
|  | 25 - 29 | 7.4 | 8.4 | 8.4 |
|  | 30 - 39 | 19.0 | 17.6 | 17.6 |
|  | 40 - 49 | 17.7 | 15.8 | 15.8 |
|  | 50 - 59 | 14.6 | 15.8 | 15.8 |
|  | 60 - 64 | 8.8 | 8.1 | 8.1 |
|  | 65 Plus | 25.2 | 22.4 | 22.4 |
| **Sex** | Male | 49.6 | 49.0 | 49.0 |
|  | Female | 50.4 | 51.0 | 51.0 |
| **Education Status** | Less than High School | 4.6 | 9.4 | 9.4 |
|  | High School Equivalent | 17.4 | 28.9 | 28.9 |
|  | College/Associate Degree | 43.2 | 26.4 | 26.4 |
|  | >= college | 34.9 | 35.4 | 35.4 |
| **Race/Ethnicity** | Non-Hispanic White | 64.1 | 61.3 | 61.3 |
|  | Non-Hispanic Black | 11.7 | 12.1 | 12.1 |
|  | Hispanic | 17.4 | 17.5 | 17.5 |
|  | All other | 6.8 | 9.1 | 9.1 |
