# Supplementary material for: Religious values and confidence in science: Perceived tensions and common ground
Source: PLoS One. 2025 Sep 19;20(9):e0332477. doi: 10.1371/journal.pone.0332477 (PMC12448960; doi:10.1371/journal.pone.0332477)
Supplement: S2 Table — (DOCX) [file pone.0332477.s003.docx]

**S2 Table. Bivariate Correlations for Key Independent Variables and Dependent Variable for Study 1.**

|  | (1) | (2) | (3) | (4) |
| --- | --- | --- | --- | --- |
| (1) Confidence in science | 1.00 |  |  |  |
| (2) Importance of religion | -0.15*** | 1.00 |  |  |
| (3) Conflict of religion and science | -0.26*** | 0.64*** | 1.00 |  |
| (4) Religious-moral values | -0.25*** | 0.56*** | 0.58*** | 1.00 |

*** p < .001
