# Supplementary material for: Religious values and confidence in science: Perceived tensions and common ground
Source: PLoS One. 2025 Sep 19;20(9):e0332477. doi: 10.1371/journal.pone.0332477 (PMC12448960; doi:10.1371/journal.pone.0332477)
Supplement: S3 Table — (DOCX) [file pone.0332477.s004.docx]

**S3 Table. VIF and Tolerance Values for Study 1.**

|  | Model 1 | | Model 2 | | | Model 3 | | |  |
| --- | --- | --- | --- | --- | --- | --- | --- | --- | --- |
|  | Tolerance | VIF | | Tolerance | VIF | | Tolerance | VIF | |
| Sex | 0.93 | 1.07 | | 0.91 | 1.10 | | 0.89 | 1.12 | |
| Age | 0.90 | 1.11 | | 0.85 | 1.18 | | 0.85 | 1.18 | |
| Education | 0.93 | 1.08 | | 0.90 | 1.11 | | 0.89 | 1.12 | |
| Black (reference category: White) | 0.92 | 1.08 | | 0.86 | 1.16 | | 0.86 | 1.16 | |
| Hispanic (reference category: White) | 0.92 | 1.09 | | 0.91 | 1.10 | | 0.90 | 1.10 | |
| Other races (reference category: White) | 0.94 | 1.06 | | 0.94 | 1.07 | | 0.93 | 1.07 | |
| Ideology (conservative) | 0.95 | 1.05 | | 0.84 | 1.19 | | 0.78 | 1.28 | |
| Importance of religion |  |  | | 0.55 | 1.83 | | 0.50 | 2.00 | |
| Conflict of religion and science: Whenever science and religion conflict, religion is always right. |  |  | | 0.52 | 1.91 | | 0.48 | 2.08 | |
| Religious-moral values |  |  | |  |  | | 0.54 | 1.86 | |
