# Supplementary material for: Religious values and confidence in science: Perceived tensions and common ground
Source: PLoS One. 2025 Sep 19;20(9):e0332477. doi: 10.1371/journal.pone.0332477 (PMC12448960; doi:10.1371/journal.pone.0332477)
Supplement: S4 Table — (DOCX) [file pone.0332477.s005.docx]

**S4 Table. Bivariate Correlations for Key Independent Variables and Dependent Variable for Study 2.**

|  | (1) | (2) | (3) | (4) |
| --- | --- | --- | --- | --- |
| (1) Confidence in science | 1.00 |  |  |  |
| (2) Importance of religion | -.10** | 1.00 |  |  |
| (3) Conflict of religion and science | -.22*** | .28*** | 1.00 |  |
| (4) Identifying science as in line with religion | .24*** | .41*** | .08* | 1.00 |

* p < .05, *** p < .001
