# Supplementary material for: Religious values and confidence in science: Perceived tensions and common ground
Source: PLoS One. 2025 Sep 19;20(9):e0332477. doi: 10.1371/journal.pone.0332477 (PMC12448960; doi:10.1371/journal.pone.0332477)
Supplement: S5 Table — (DOCX) [file pone.0332477.s006.docx]

**S5 Table. VIF and Tolerance Values for Study 2.**

|  | Model 1 | | Model 2 | | | Model 3 | | |  |
| --- | --- | --- | --- | --- | --- | --- | --- | --- | --- |
|  | Tolerance | VIF | | Tolerance | VIF | | Tolerance | VIF | |
| Sex | 0.98 | 1.02 | | 0.96 | 1.04 | | 0.96 | 1.04 | |
| Age | 0.90 | 1.11 | | 0.88 | 1.14 | | 0.88 | 1.14 | |
| Education | 0.86 | 1.16 | | 0.86 | 1.17 | | 0.85 | 1.17 | |
| Black (reference category: White) | 0.93 | 1.08 | | 0.84 | 1.19 | | 0.84 | 1.19 | |
| Hispanic (reference category: White) | 0.92 | 1.09 | | 0.89 | 1.13 | | 0.88 | 1.13 | |
| Other races (reference category: White) | 0.89 | 1.13 | | 0.88 | 1.13 | | 0.88 | 1.14 | |
| Ideology (conservative) | 0.96 | 1.04 | | 0.76 | 1.31 | | 0.76 | 1.32 | |
| Importance of religion |  |  | | 0.74 | 1.35 | | 0.63 | 1.59 | |
| Conflict of religion and science: Science is incompatible with my religious views. |  |  | | 0.82 | 1.22 | | 0.82 | 1.22 | |
| Identifying science as in line with religion |  |  | |  |  | | 0.82 | 1.22 | |
