# Supplementary figures and images for: Religious values and confidence in science: Perceived tensions and common ground
Source: PLoS One. 2025 Sep 19;20(9):e0332477. doi: 10.1371/journal.pone.0332477 (PMC12448960; doi:10.1371/journal.pone.0332477)

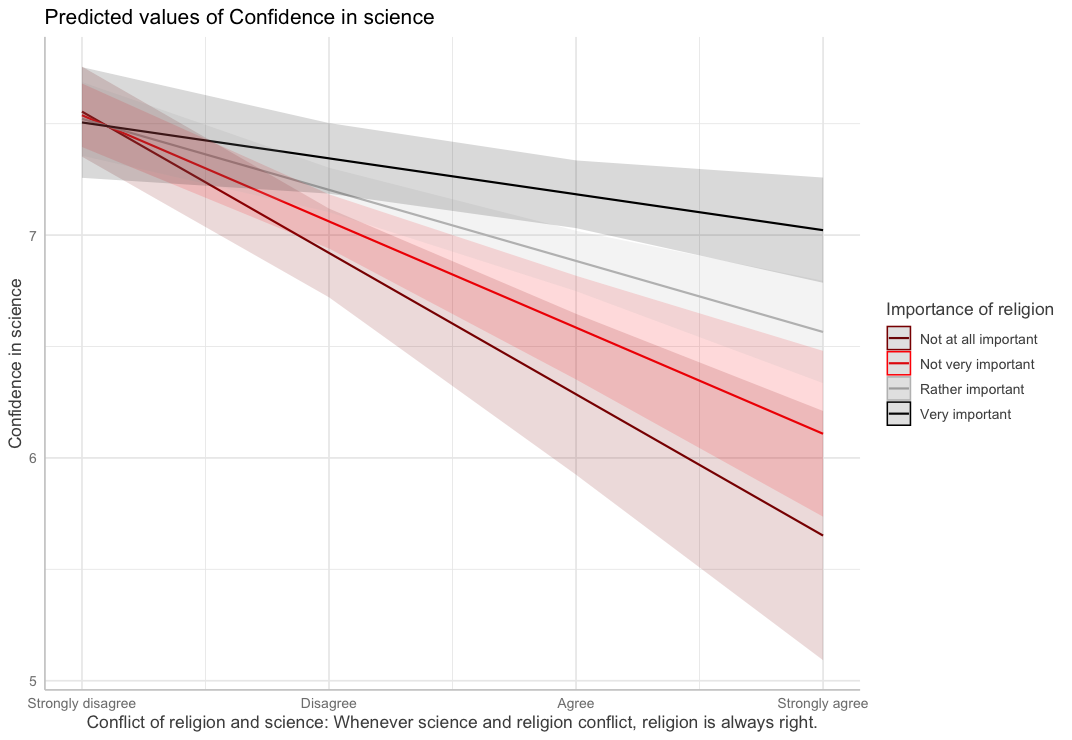

Supplement: S1 Fig — (TIFF) [file pone.0332477.s007.tiff]
